# Case Report: Next generation sequencing identifies a *NAB2*-*STAT6* fusion in Glioblastoma

**DOI:** 10.1186/s13000-016-0455-9

**Published:** 2016-01-27

**Authors:** Phedias Diamandis, Ruben Ferrer-Luna, Raymond Y. Huang, Rebecca D. Folkerth, Azra H. Ligon, Patrick Y. Wen, Rameen Beroukhim, Keith L. Ligon, Shakti H. Ramkissoon

**Affiliations:** Neuropathology Program, Department of Laboratory Medicine and Pathobiology, University of Toronto, 27 King’s College Circle, Toronto, ON M5S Canada; Department of Pathology, Brigham and Women’s Hospital, 75 Francis Street, Boston, MA 02115 USA; Cancer Program, Broad Institute of MIT and Harvard, 415 Main St, Cambridge, MA 02142 USA; Department of Cancer Biology, Dana-Farber Cancer Institute, 450 Brookline Avenue, Boston, MA 02115 USA; Department of Radiology, Brigham and Women’s Hospital, 75 Francis Street, Boston, MA 02115 USA; Department of Pathology, Harvard Medical School, 25 Shattuck Street, Boston, MA 02115 USA; Department of Pathology, Boston Children’s Hospital, 300 Longwood Avenue, Boston, MA 02115 USA; Department of Medical Oncology, Dana-Farber Cancer Institute, 450 Brookline Avenue, Boston, MA 02115 USA

**Keywords:** NAB2-STAT6, Glioblastoma, Next generation sequencing

## Abstract

**Background:**

Molecular profiling has uncovered genetic subtypes of glioblastoma (GBM), including tumors with *IDH1* mutations that confer increase survival and improved response to standard-of-care therapies.  By mapping the genetic landscape of brain tumors in routine clinical practice, we enable rapid identification of targetable genetic alterations.

**Case Presentation:**

A 29-year-old male presented with new onset seizures prompting neuroimaging studies, which revealed an enhancing 5 cm intra-axial lesion involving the right parietal lobe. He underwent a subtotal resection and pathologic examination revealed glioblastoma with mitoses, microvascular proliferation and necrosis. Immunohistochemical (IHC) analysis showed diffuse expression of GFAP, OLIG2 and SOX2 consistent with a tumor of glial lineage. Tumor cells were positive for IDH1(R132H) and negative for ATRX. Clinical targeted-exome sequencing (DFBWCC Oncopanel) identified multiple functional variants including *IDH1* (p.R132H), *TP53* (p.Y126_splice), *ATRX* (p.R1302fs*), *HNF1A* (p.R263H) and *NF1* (p.H2592del) variants and a *NAB2-STAT6* gene fusion event involving *NAB2* exon 3 and *STAT6* exon 18. Array comparative genomic hybridization (aCGH) further revealed a focal amplification of *NAB2* and *STAT6*.  IHC analysis demonstrated strong heterogenous STAT6 nuclear localization (in 20 % of tumor cells).

**Conclusions:**

While *NAB2:STAT6* fusions are common in solitary fibrous tumors (SFT), we report this event for the first time in a newly diagnosed, secondary-type GBM or any other non-SFT. Our study further highlights the value of comprehensive genomic analyses in identifying patient-specific targetable mutations and rearrangements.

## Background

Glioblastoma (GBM) is the most common, primary malignant brain tumor of adults with a median survival of 15 months despite multimodal surgical, chemo and radiotherapy [1]. GBMs are characterized by infiltrating tumor cells with nuclear pleomorphism, mitotic activity and accompanying necrosis and/or endothelial proliferation. More recently, molecular profiling has uncovered genetic subtypes among histologically indistinguishable GBMs that confer increased survival and improved response to standard-of-care therapies. For example, tumors with *isocitrate dehydrogenase* (*IDH1*/*2*) mutations show prolonged survival while tumors with *MGMT* promoter methylation benefit from temozolomide therapy [2, 3]. Despite inclusion of clinically relevant molecular events in routine diagnostic evaluation of brain tumors, molecular testing has largely remained limited to a small number of genetic alterations linked to specific tumor subtypes (e.g., 1p/19q co-deletion in oligodendrogliomas). Such focused interrogation strategies however ignore the genetic heterogeneity of cancer and prevent screening for less common, yet clinically actionable, genetic events. Indeed, assessment of *IDH1* mutation status by immunochemistry only identifies the most common *IDH1* R132H alteration, while other less frequent, yet prognostically important *IDH1*/*2* are not assessed. Furthermore, other rare genetic changes that are potentially therapeutically actionable, such as *BRAF* (*p*.V600E) mutations, are not routinely profiled in high-grade gliomas [4]. Comprehensive genomic analysis of brain tumors provides objective companion data to support and refine histologic diagnoses [5]. Furthermore, by mapping the genetic landscape of brain tumors in routine clinical practice, we enable rapid identification of targetable genetic alterations and support patient enrollment onto molecularly stratified clinical trials.

## Case presentation

**Fig. 1:**
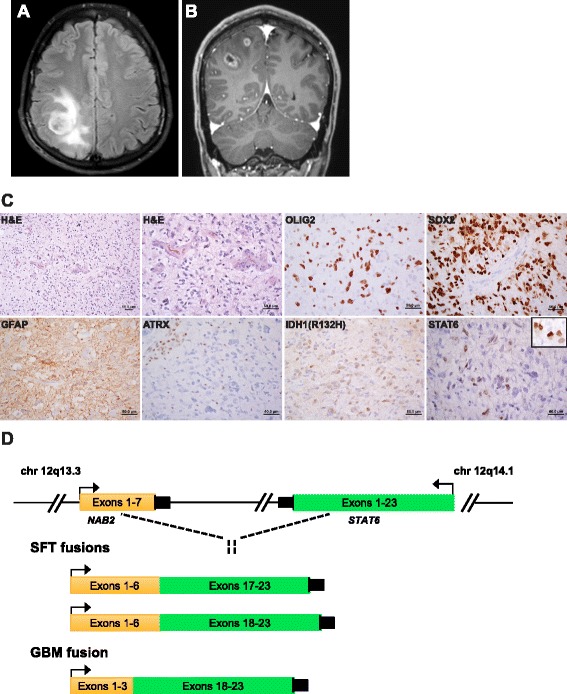
**a** Coronal T2 FLAIR highlights the intra-parenchymal location of the complex tumor resection bed and associated edema. **b** Axial post contrast T1 MRI showing peripheral ring enhancement and progression from previous post-surgical imaging (right). **c** H&E sections showing a hypercellular fibrillary neoplasm with morphological atypia, mitoses, and endothelial proliferation. Necrosis was also present (not shown). The tumor showed diffuse expression of the glial markers Olig2, Sox2 and GFAP. Immunoreactivity with antibody against the R132H IDH1 mutation. ATRX immunostaining highlighting loss within the tumor and normal retained expression in a adjacent vessel. Immunohistochemistry with the STAT6 antibody showing strong nuclear expression in 15–20 % of tumor nuclei (inset) consistent with previous described function of *NAB2*-*STAT6* fusion event. **d** Schematic demonstrating the locations of common *NAB2*-*STAT6* rearrangements in solitary fibrous tumor (SFTs), the presented case GBM and other *STAT6* rearrangements reported in GBMs

A previously healthy 29-year-old male presented to an outside hospital with a symptomatic intra-axial enhancing right parietal brain lesion necessitating surgical management and adjuvant temozolomide (Fig. 1a, b). Histology showed a densely cellular infiltrating glial neoplasm comprised of severely atypical cells with mitoses, vascular proliferation and necrosis consistent with GBM (Fig. 1c). Immunohistochemistry demonstrates tumor cells were positive for GFAP, OLIG2, SOX2, IDH1(R132H) and a MIB1 proliferation index of >30 % (Fig. 1c). *MGMT* methylation testing revealed that the tumor was positive for promoter methylation.

Targeted exome sequencing (OncoPanel) of 300 cancer-associated genes and 113 introns from 35 genes for rearrangement was performed on DNA extracted from formalin-fixed paraffin embedded tumor tissue [6, 7]. OncoPanel revealed *IDH1* (*p.R132H*), *TP53* (*p.Y126*_*splice*), *ATRX* (p.R1302fs*), *HNF1A* (*p.R263H*) and *NF1* (*p.H2592del*) variants, several variants of unknown significance, and a *NAB2*-*STAT6* fusion involving exon 3 of *NAB2* and exon 18 of *STAT6* (Fig. 1d, Table 1). This rearrangement has not previously been reported outside the context of solitary fibrous tumors (SFTs). In SFTs, fusion products are variable, but typically involve exons 6/7 of *NAB2* and exons 17/18 of *STAT6* [8] (Fig. 1d). Similar to the functional consequence of this fusion product in SFTs, we confirmed that this novel *NAB2*-*STAT6* fusion detected in our GBM patient resulted in strong STAT6 nuclear localization [9] (Santa Cruz, catalog #sc-621), however this was present in only a subpopulation of cells (Fig. 1c).

**Table 1:** Mutations identified in GBM patient by targeted exome sequencing (OncoPanel)

Gene Symbol	Protein Change	Allelic Fraction
*IDH1*	*p.R132H*	*0.50*
*TP53*	*p.Y126_splice*	*0.85*
*ATRX*	*p.R1302fs*	*0.46*
*HNF1A*	*p.R263H*	*0.16*
*NF1*	*p.H2592del*	*0.38*
*GLI1*	*p.G798R*	*0.73*
*MLH1*	*p.R389Q*	*0.46*
*MLL2*	*p.Q4557_splice*	*0.36*

To determine whether this event might be recurrent in gliomas, and given multiple genomic alterations detected in this tumor closely resemble those found in adult low-grade gliomas (ALGGs) (mutations in *IDH1*, *TP53* and *ATRX*) [10], we investigated the frequency of NAB2-STAT6 fusions in The Cancer Genome Atlas (TCGA) low-grade glioma database (http://cancergenome.nih.gov/). This analysis included 50 tumors with whole genome sequencing (WGS) and 311 tumors with whole exome sequences (WES). Among this 361 patient cohort, we did not detect any evidence of *NAB2*-*STAT6* fusions, nor fusion events involving *NAB2* or *STAT6* with other fusion partners.

We next analyzed TCGA adult GBM datasets including 42 and 164 tumors tested by WGS and RNA sequencing, respectively. Similar to our findings among ALGGs, we did not detect *NAB2*-*STAT6* fusions but did identify two unique *STAT6* fusion events *STAT6*-*CPM* and *HIPK2*-*STAT6* and each co-amplified with oncogenes *CDK4* and *MDM2* in the amplicon on chromosome 12q13-15. These observations suggest that *STAT6* fusions occur at a low frequency but may be recurrent with other 12q13-15 amplification events.

Given this finding, we performed genome wide array comparative genomic hybridization (aCGH), or copy number analysis, of our case study GBM and also identified co-amplification of *NAB2*, *STAT6* and *CDK4* involving a 12q3-14 amplicon of 0.6 Mb (Table 2).

**Table 2:** aCGH copy number alterations identified in GBM patient

*Gene/Region*	*Chromosome Band*	*Copy Number Change*	*Nucleotides (GRCh37//hg19)*
*MYCL1*	*1p34.2*	*--*	*--*
*CDKN2C*	*1p33*	*--*	*--*
*PIK3C2B*	*1q32.1*	*--*	
*MDM4*	*1q32.1*	*--*	*--*
*AKT3*	*1q44*	*--*	
*MYCN*	*2p24.3*	*--*	*--*
*PIK3CA*	*3q26.32*	*--*	
*SOX2*	*3q26.33*	*--*	*--*
*FGFR3*	*4p16.3*	*--*	
*PDGFRA*	*4q12*	*550 kb focal gain*	*chr4:55,072,465–55,622,596*
*TERT*	*5p15.33*	*--*	
*MYB*	*6q23.3*	*--*	*--*
*PARK2*	*6q26*	*--*	
*QKI*	*6q26*	*--*	*--*
*EGFR*	*7p11.2*	*--*	
*EGFRvIII*	*7p11.2*	*--*	*--*
*CDK6*	*7q21.2*	*--*	
*MET*	*7q31.2*	*--*	*--*
*BRAF*	*7q34*	*--*	
*FGFR1*	*8p11 23-p11.22 *	*--*	*--*
*MYBL1*	*8q13.1*	*--*	
*MYC*	*8q24.21*	*--*	*--*
*CDKN2A*	*9p21.3*	*38.5 Mb single copy gain*	*chr9:204,104–38,731,432*
*NTRK2*	*9q21.32-q21.33*	*1.3 Mb focal gain*	*chr9:86,840,129–88,134,100*
*NTRK2*	*9q21.32-q21.33*	*159 kb intragenic amplification*	*chr9:87,344,078–87,503,027*
*PTEN*	*10q23.31*	*82.6 Mb single copy loss*	*chr10:52,805,936–135,435,714*
*FGFR2*	*10q26.13*	*82.6 Mb single copy loss*	*chr10:52,805,936–135,435,714*
*CCND2*	*12p13.32*	*34.6 Mb single copy loss*	*chr12:163,593–34,756,209*
*NAB2*	*12q13.3*	*37 kb amplification*	*chr12:57,484,461 –57,521,151*
*STAT6*	*12q13.3*	*37 kb amplification*	*chr12:57,484,461 –57,521,151*
*CDK4*	*12q14.1*	*131 kb amplification*	*chr12:58,034,214–58,165,540*
*MDM2*	*12q15*	*95.3 Mb single copy loss*	*chr12:38,448,667–133,779,076*
*RB1*	*13q14.2*	*Single copy loss via monosomy 13*	*chr13:19,296,544–115,105,297*
*TP53*	*17p13.1*	*19.5 Mb single copy loss*	*chr17:47,546–19,536,368*
*NF1*	*17q11.2*	*--*	
*SMARCB1/INI1 *	*22q11.23*	*Single copy loss via monosomy 22*	*chr22:16,133,474–51,219,009*
*NF2*	*22q12.2*	*Single copy loss via monosomy 22*	*chr22:16,133,474–51,219,009*
*1p-*	*n/a*	*--*	*--*
*4p-*	*n/a*	*--*	
*Monosomy 6*	*n/a*		*--*
*6q-*	*n/a*	*--*	
*Polysomy 7*	*n/a*		*--*
*7p-*	*n/a*	*--*	
*Monosomy 10*	*n/a*		*--*
*10q-*	*n/a*	*82.6 Mb single copy loss*	*chr10:52,805,936–135,435,714*
*11p-*	*n/a*	*--*	*--*
*Monosomy 14*	*n/a*	*--*	
*idic(17p11.2)*	*n/a*		*--*
*18q-*	*n/a*	*--*	
*19q-*	*n/a*		*--*
*Monosomy 22*	*n/a*	*Detected*	*chr22:16,133,474–51,219,009*

## Conclusions

Incorporating molecular analyses into current diagnostic pipelines for GBM patients may provide an avenue for identifying novel aberrations or events that have been previously reported in other tumor types [7]. One study reported that such a genome wide screening strategy can in fact yield candidate actionable genetic alterations in every case analysed [6]. Here we demonstrate that targeted-exome sequencing of a GBM revealed a *NAB2*-*STAT6* fusion, which is a known oncogenic driver in SFTs but has not been reported in other cancers. In SFTs, the novel fusion product results in nuclear translocation of the STAT6 transcriptional activating domain and activation of the early growth response (EGR1) pathway leading to tumorigensis [8, 11]. The strong nuclear STAT6 staining in a subpopulation of GBM cells parallels what is found in SFTs, suggesting a similar mechanism of action. Given its high frequency in SFTs, the *NAB2*-*STAT6* fusion has raised interest as both a diagnostic and potentially druggable therapeutic target [12]. Although the frequency of this event has not been fully explored, our results from querying TCGA ALGG and GBM datasets demonstrates that specific *NAB2*-*STAT6* fusion events are rare in gliomas but that STAT6 fusions are recurrent events with several partners in adult GBM. Despite the low recurrence frequency, this fusion supports repurposing drugs developed against the *NAB2*-*STAT6* or the EGR pathway in SFTs as a potential alternative or adjuvant therapy for patients with genetically similar gliomas.

Interestingly, similar to other *STAT6* rearrangement events in GBMs, we noted *CDK4* amplification in this patient. Chromosome 12 is frequently subject to a storm of amplification/rearrangement events in GBMs, including *CDK4*, *MDM2*, and *HMGA2* amplifications as well as other regions (including *KRAS*). These events suggest a more complex rearrangement involving much of the chromosome and are reminiscent of ring chromosomes found in dedifferentiated liposarcomas [13–15]. Given the genetic proximity of *CDK4* and *MDM2* to *STAT6* and *NAB2* in the chromosomal region 12q13-15, the fusion product may represent a consequence of this more complex rearrangement. Understanding the precise functional consequences of this molecular alteration in glioma biology will be important to guiding future therapies of similar cases [13]. As genome wide sequencing strategies become more widely available in the clinical setting, infrequent mutations and/or rare fusion events may collectively represent a brain tumor patient population that can be managed with targeted therapies approved for use in other tumor types. This strategy may pave the way for improving outcomes in a small subset of GBM patients.

### Consent

Written informed consent was obtained for 10–417 and 11–104 (OncoPanel) from the patient for sequencing analysis, publication of this report and accompanying images.

## Abbreviations

aCGH: array comparative genomic hybridization
ALGG: adult low grade glioma
EGR1: early growth response
GBM: glioblastoma
SFT: solitary fibrous tumor
TCGA: the cancer genome atlas
